# Superior Induction of Apoptosis by Blue Light Compared to Red Light Photodynamic Therapy in Cutaneous Squamous Cell Carcinoma Keratinocytes

**DOI:** 10.1002/jbio.70272

**Published:** 2026-05-15

**Authors:** Julia Stolyar, Margaret Kabakova, Paras Patel, Julie Saadia‐Hazkour, Evan Austin, Jared Jagdeo

**Affiliations:** ^1^ Department of Dermatology, State University of New York Downstate Health Sciences University Brooklyn New York USA; ^2^ Dermatology Services, Veterans Affairs New York Harbor Healthcare System – Brooklyn Campus Brooklyn New York USA; ^3^ Rowan University School of Osteopathic Medicine Stratford New Jersey USA

**Keywords:** actinic keratosis, blue light, photodynamic therapy, red light, squamous cell carcinoma

## Abstract

This study compared apoptosis induction in squamous cell carcinoma keratinocytes following exposure to 5‐aminolevulinic acid combined with either blue light (417 ± 5 nm) or red light (635 ± 5 nm) photodynamic therapy, with apoptotic activity quantified via levels of Annexin V expression. Our results demonstrated that blue light induces superior levels of apoptosis in squamous cell carcinoma keratinocytes compared to red light at the parameters tested. Both wavelengths demonstrate additional dose‐ and temperature‐dependent effects across two squamous cell carcinoma cell lines. These conclusions had significant implications for the clinical selection of a light source in combination with 5‐aminolevulinic acid photodynamic therapy in the treatment of actinic keratosis and early squamous cell carcinoma lesions.

## Graphical abstract

This study demonstrates that 5‐ALA BL PDT induces greater levels of apoptosis in SCC cells in a wavelength‐, 5‐ALA dose‐, and temperature‐dependent manner, compared to 5‐ALA RL PDT, with higher concentrations of ALA and elevated temperatures stimulating the greatest apoptotic activity.

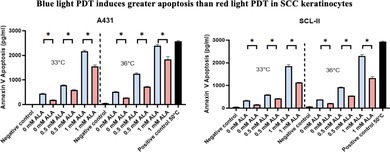

**Abbreviations**
5‐ALA: 5‐aminolevulinic acid
AK: actinic keratosis
BL: blue light
PDT: photodynamic therapy
PP‐IX: protoporphyrin IX
PS: phosphatidylserine
RL: red light
ROS: reactive oxygen species
SCC: squamous cell carcinoma

## 1 Introduction

Actinic keratoses (AKs) are precancerous epidermal skin lesions that develop as a result of cumulative ultraviolet damage from the sun [1]. AKs and squamous cell carcinoma (SCC) are lesions that arise from aberrant keratinocyte proliferation, with AKs characterized by a less aggressive clinical phenotype [2]. Approximately 10% of AKs progress into SCC over the course of 2 years, making their effective management critical for public health [3]. AK is among the most frequently diagnosed dermatologic conditions, with a prevalence rate of 14% globally [4]. SCC ranks as the second most common skin cancer in the United States after basal cell carcinoma, with over 2.5 million cases and approximately 13 000 related deaths annually worldwide [5, 6, 7]. National costs associated with treatment of AKs exceed $1.2 billion annually [8]. Within the Veterans Affairs healthcare system, where this study was conducted, AK and SCC represent a substantial burden, with 200 000 veterans diagnosed with AK and more than 25 000 diagnosed with SCC annually, accounting for over $350 million in healthcare expenditures each year [9].

AKs are commonly treated with photodynamic therapy (PDT), a non‐invasive, cost‐effective therapeutic modality in which a photosensitizer, most commonly 5‐aminolevulinic acid (5‐ALA) or methyl aminolevulinate (MAL), undergoes photoactivation alongside oxygen [10, 11]. 5‐ALA PDT represents an important treatment option due to its efficacy in treating multiple AK lesions concurrently [12]. Mechanistically, AK and SCC cells have decreased expression of the enzyme ferrochelatase, which normally converts 5‐ALA into heme [13, 14]. Therefore, these cells preferentially convert and accumulate the photoactive compound protoporphyrin IX (PP‐IX), relative to normal cells [13, 14]. Upon exposure to a light source, excitation of PP‐IX generates reactive oxygen species (ROS), leading to apoptosis and cellular death. PDT efficacy is measured by its effectiveness in killing the targeted aberrant keratinocytes comprising AKs and thus clearing the AK lesions. Controversy still remains concerning which light source is more effective at killing aberrant keratinocytes when combined with 5‐ALA [15, 16]. It has been established that PP‐IX has a greater peak for BL than for RL. [17, 18] However, to our knowledge, no other in vitro studies have directly compared wavelength efficacy in AK and SCC cell models.

The two predominant commercially available PDT light sources emit either blue (417 ± 5 nm) or red (635 ± 5 nm) wavelengths. Red light (RL) penetrates deeper into the dermis compared to blue light (BL), enabling it to exert its effects on deeper tumor regions, though this increased depth is less clinically relevant for superficial epidermal lesions, including AKs and SCC in situ [19]. A previous study in human cancer keratinocytes suggested superior ROS generation and apoptosis induction by BL PDT compared to RL PDT, potentially due to higher PP‐IX absorption at 410 nm [17]. PP‐IX has an absorption spectrum that includes 5 peaks at 410 nm, 510 nm, 545 nm, 580 nm, and 630 nm. PP‐IX is preferentially absorbed at 410 nm, which favors BL over RL. [18] Radiometric testing comparing the two wavelengths showed that BL achieves the greatest effective irradiance, resulting in optimal PP‐IX absorption at the skin surface, but limited effect at a depth of 500 μm [20]. In contrast, RL shows reduced absorption of PP‐IX, yet maximal effective fluence is reached as penetration depth increases [20].

This study seeks to directly quantify and compare apoptosis induction in SCC keratinocytes following exposure to 5‐ALA combined with either BL or RL PDT. Determining the superior PDT modality could significantly impact clinical practice by guiding optimized PDT choices, thereby improving patient outcomes and resource allocation.

## 2 Materials and Methods

### 2.1 Materials

A431 and SCL‐II cell lines were obtained from Cytion (Heidelberg, Germany). The 5‐ALA compound was procured from Sun Pharmaceuticals (Wilmington, MA). Dulbecco's Modified Eagle's Medium (DMEM) was acquired from Sigma‐Aldrich (St. Louis, MO). Phosphate‐buffered saline (PBS), trypsin, and Antibiotic‐Antimycotic (ABAM) (100x) were sourced from Thermo Fisher Scientific (Waltham, MA). Fetal bovine serum (FBS) was obtained from Atlanta Biologicals (Flowery Branch, Georgia). 6‐well plates were procured from Corning (Corning, New York). Blue‐light irradiation was delivered using the commercially available BLU‐U device from Sun Pharmaceuticals (Wilmington, MA), emitting at 417 ± 5 nm, while red‐light irradiation was delivered using the RhodoLED device from Biofrontera (Woburn, MA), emitting at 635 ± 5 nm. Annexin‐V ELISA kits were obtained from Abcam (Cambridge, UK) to quantify apoptosis.

### 2.2 Cell Culture

Two SCC cell lines, A431 and SCL‐II, were cultured with medium composed of 4.5 g/L glucose DMEM supplemented with 10% FBS and 1% antibiotic‐antimycotic. Cells were kept in a humidified incubator set to 37°C with 5% CO_2_. Cells were seeded at a density of 50 000 cells per well in 6‐well plates and incubated for 24 h prior to experimental interventions. All experimental procedures were conducted using cell cultures at passage 12 or lower.

### 2.3 ALA Treatment and PDT

Cells were treated with 0, 0.5, or 1 mM 5‐ALA solutions freshly prepared in serum‐free culture medium immediately prior to use. After 5‐ALA application, cells were incubated at controlled temperatures (33°C and 36°C) for 30 min and shielded from ambient light [21, 22]. For each experimental condition, appropriate negative (0 ALA, no light), positive (heat‐induced apoptosis, no light, 50°C), and light‐only controls (blue or red‐light exposure with 0 ALA) were established.

### 2.4 Blue and Red Light Photoactivation

After ALA incubation, cells were irradiated using either the BL or RL device. BL irradiation was conducted with the BLU‐U device—emitting wavelengths of 417 ± 5 nm—for 1000 s at a power density of 10 mW/cm^2^, achieving a fluence of 10 J/cm^2^. RL irradiation was performed using the RhodoLED device—emitting wavelengths of 635 ± 5 nm—for 600 s at 61 mW/cm^2^, achieving a fluence of 37 J/cm^2^. These irradiation parameters align with FDA‐approved clinical PDT protocols.

### 2.5 Cell Harvesting and Apoptosis Quantification

Following PDT treatment, cells were detached using trypsin–EDTA, neutralized with 4.5 g/L glucose DMEM supplemented with 10% FBS and 1% antibiotic‐antimycotic, centrifuged, and prepared for apoptosis assays. Apoptosis was quantified using the Annexin V ELISA kit according to the manufacturer's instructions, with absorbance measured using a 96‐well plate reader with an absorbance wavelength of 450 nm. Experimental conditions were replicated to ensure reproducibility and statistical significance.

Phosphatidylserine (PS) is a phospholipid that normally resides within the inner cell membrane. During apoptosis, PS externalization occurs, allowing PS to be utilized as a marker of early cell death [21]. Annexin V is a calcium‐dependent protein with a high affinity for PS and can thus be used to identify cells that have begun to undergo apoptosis [21]. As such, Annexin V ELISA is an appropriate measure of apoptotic activity in SCC cell cultures.

### 2.6 Statistical Analysis

Two‐tailed Student's *t*‐tests, conducted in GraphPad Prism, were used to compare the ELISA results of BL and RL PDT groups at respective ALA concentrations and temperatures and their respective controls. For the ELISA assay, the sample size was *n* = 3, with the experimental protocol repeated twice using two different cell lines. A significance level of α < 0.05 was applied.

## 3 Results and Discussion

### 3.1 Results

**FIGURE 1:**
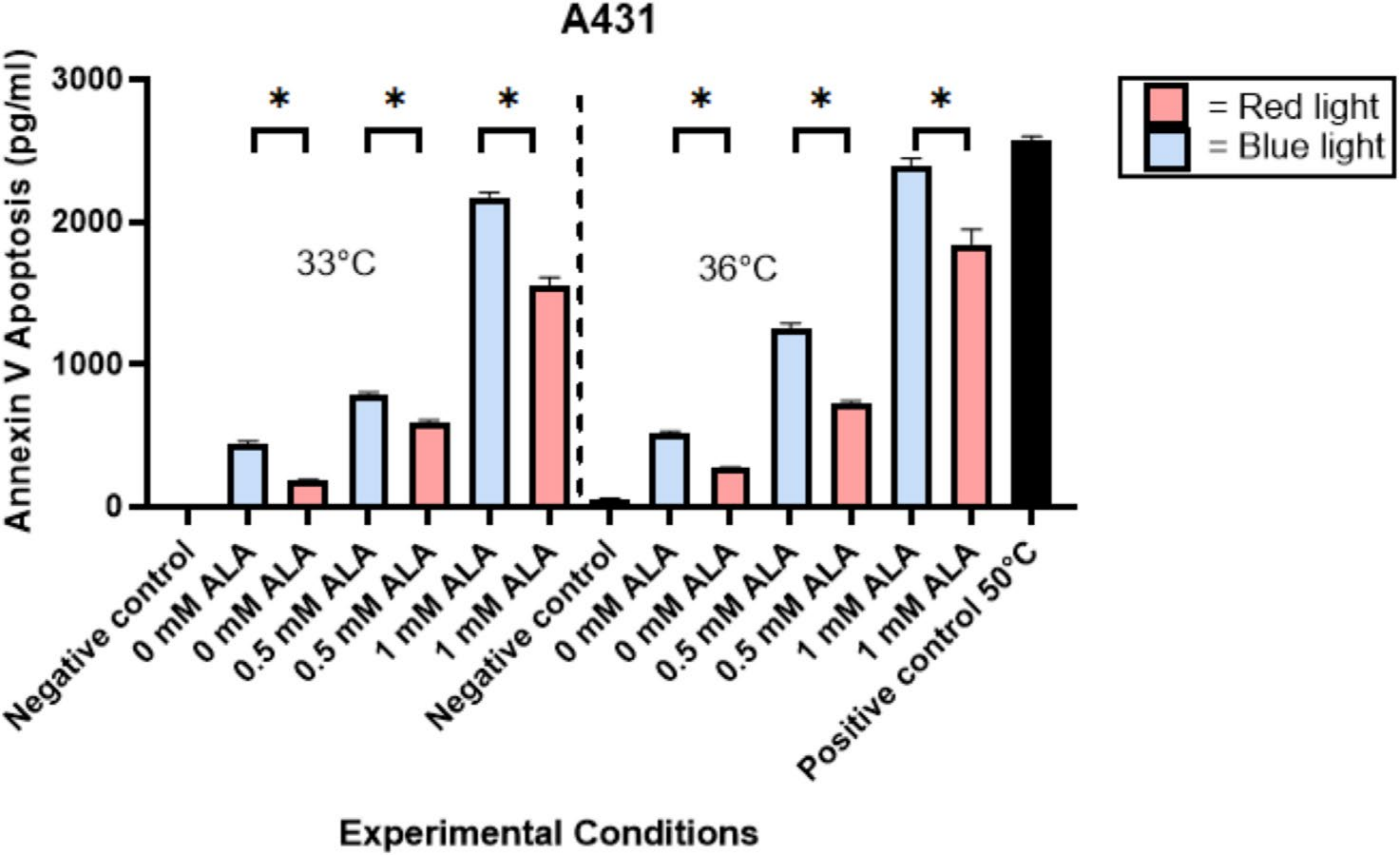
Annexin V levels in A431 squamous cell carcinoma cells. A431 cells were irradiated with BL at a dose of 10 J/cm^2^ or with RL at a dose of 37 J/cm^2^. Apoptosis was quantified via Annexin V ELISA. A t‐test compared Annexin V production between BL‐treated samples, RL‐treated samples, and controls (*n* = 3, *p* < 0.05). Abbreviations: ALA, aminolaevulinic acid; BL, blue light; RL, red light; 33C, 33 degrees Celsius; 36C, 36 degrees Celsius; *, statistically significant. Error bars represent standard error of the mean.

**FIGURE 2:**
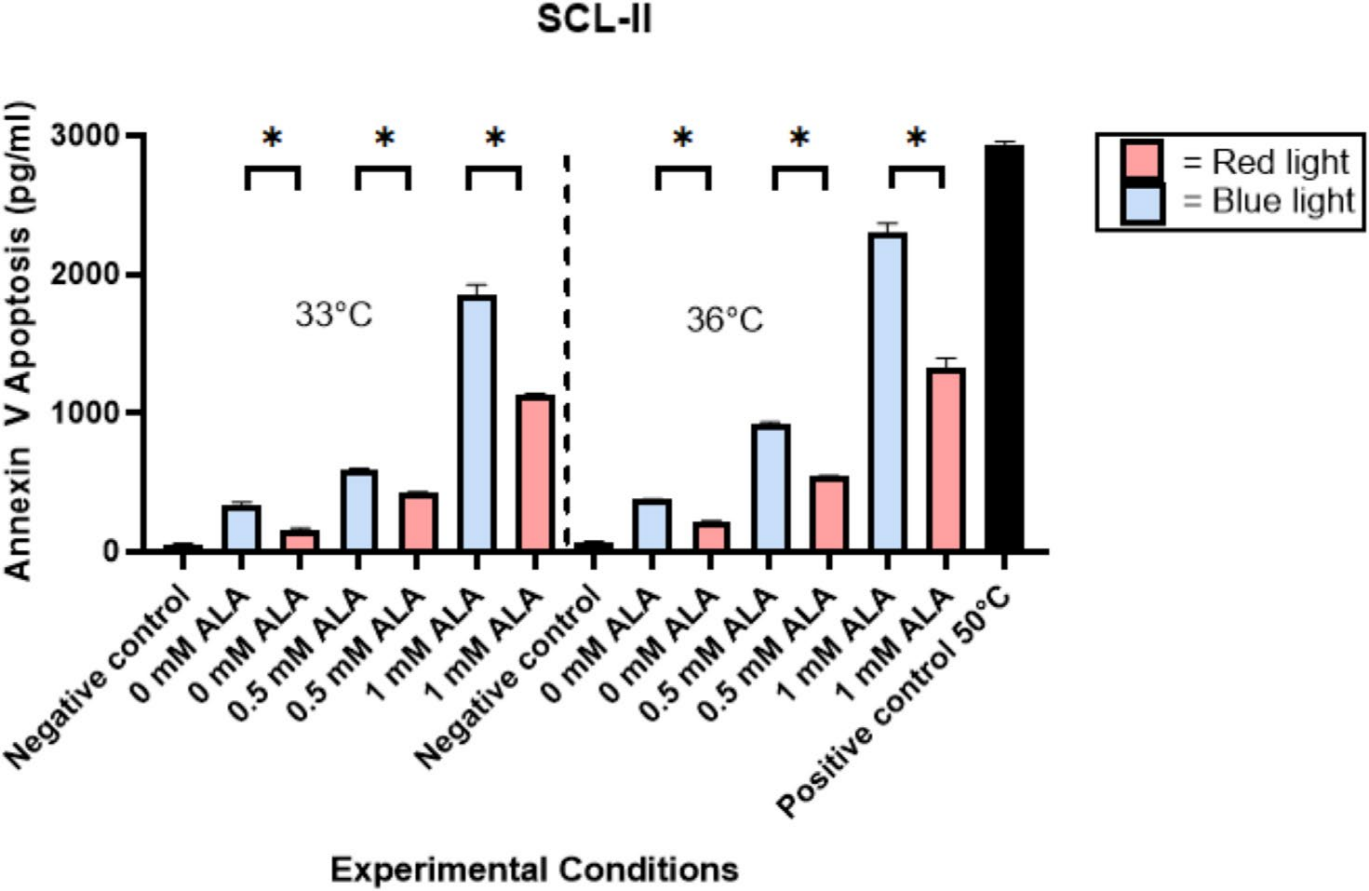
Annexin V levels in SCL‐II squamous cell carcinoma cells. SCL‐II cells were irradiated with BL at a dose of 10 J/cm^2^ or with RL at a dose of 37 J/cm^2^. Apoptosis was quantified via Annexin V ELISA. A t‐test compared Annexin V production between BL‐treated samples, RL‐treated samples, and controls (*n* = 3, *p* < 0.05). Abbreviations: ALA, aminolaevulinic acid; BL, blue light; RL, red light; 33C, 33 degrees Celsius; 36C, 36 degrees Celsius; *, statistically significant. Error bars represent standard error of the mean.

Irradiation of A431 and SCL‐II cells with BL at a dose of 10 J/cm^2^ resulted in greater apoptosis compared to irradiation of these cell lines with RL at a dose of 37 J/cm^2^, as confirmed by ELISA (Figures 1 and 2). These differences observed between BL and RL treatments of SCC cells were statistically significant, with *p* < 0.05 at each temperature‐ and ALA‐matched condition. BL irradiation without ALA at 33°C and 36°C exhibited increased apoptosis compared to RL. This indicates that BL alone can cause apoptosis. Both A431 and SCL‐II cells exhibited increasing Annexin V with greater concentrations of 5‐ALA, indicating that apoptosis is 5‐ALA dose‐dependent. Furthermore, at higher concentrations of 5‐ALA, the difference between levels of Annexin V produced by BL versus RL was much greater than at lower concentrations of 5‐ALA, which accounts for a synergistic effect between 5‐ALA and BL to induce increased apoptosis in SCC cells. Apoptotic activity also demonstrated temperature dependence, given that cells heated to 36°C displayed increased cell death at all conditions compared to cells heated to 33°C.

### 3.2 Discussion

Our study found that BL irradiation at a dose of 10 J/cm^2^ induces greater levels of apoptosis in SCC cells than does RL irradiation at a dose of 37 J/cm^2^. Cells irradiated with BL demonstrated greater concentrations of Annexin V than cells irradiated with RL, as measured by the ELISA, at each 5‐ALA dose‐ and temperature‐matched condition. These results support previous findings that determined BL PDT to be more efficacious at inducing cell death than RL PDT [17, 22]. Our findings can be further explained by radiometric analysis, which demonstrated that BL has a significantly greater effective irradiance of PP‐IX at the epidermal level than RL. [20] As such, BL may be particularly effective at treating superficial skin lesions, such as AKs, SCC in situ, and superficial BCC.

Beyond wavelength considerations, 5‐ALA‐mediated PDT has been known to be effective in inducing cell death, and our findings exhibited a dose‐dependent increase in apoptosis at increased concentrations of 5‐ALA, indicating that combination therapy with BL PDT and higher concentrations of ALA may result in the greatest efficacy in treating AKs and SCC in patients [23, 24]. These outcomes are consistent with earlier conclusions in our laboratory that BL‐ALA PDT induces dose‐dependent apoptosis in human dermal fibroblasts [25]. Prior AK and SCC studies observed that PDT accompanied by rising ALA concentrations stimulates apoptotic activity due to increased intracellular accumulation of PP‐IX [26, 27]. Increased PP‐IX generation may also be responsible for the temperature effects on ROS‐mediated cell death [24]. SCC cells heated at 36°C showed greater levels of Annexin V as compared to cells heated at 33°C, which is in accordance with previous findings in our laboratory that showed a statistically significant increase in apoptosis in 5‐ALA pretreated human dermal fibroblasts heated for 30 min to 36°C than those heated to 33°C [24].

Clinical data also support the efficacy of BL PDT in treating SCC in situ [28]. A retrospective study determined that BL PDT had an overall initial complete response rate of 77.9%, with the greatest response in lesions located on the face, lesions with a diameter < 2 cm, and lesions incubated in 5‐ALA for > 3 h [28]. Additionally, it was noted that recurrence only occurred in 13.2% of cases at a median of 11.7 months after treatment [28]. These findings emphasize that BL PDT is efficacious at treating superficial lesions, like SCC in situ and AK, but it is critical to factor in clinical variables such as lesion characteristics that can further influence real‐world therapeutic success [28]. Beyond triggering direct tumor cell death, PDT‐generated ROS can precipitate microvascular damage in vivo, leading to reduced tumor perfusion, and can stimulate anti‐tumor immune responses [29]. However, we did not investigate tumor vascular or immune mechanisms because our results are based on an in vitro keratinocyte model. When considering these findings together with our in vitro data regarding the wavelength‐, 5‐ALA dose‐, and temperature‐dependent apoptosis achieved by PDT, it is evident that optimization of PDT outcomes for patients requires integration of laboratory insights and clinical factors.

A strength of this study is that we tested BL versus RL exposure to cells at multiple doses of ALA and at varying temperatures. Additionally, we used commercially available phototherapy devices and manufacturer‐recommended clinical settings, which ensures standardization and relevance to clinical dermatology practice. One limitation of our experiment, as with all other in vitro studies, is that the cell culture is a monolayer and cannot replicate the complex physiologic environment found in animal and human tissue. As our study was an in vitro model, we also could not assess PDT‐associated vascular damage or immune‐mediated anti‐tumor effects; therefore, the observed apoptotic responses may not fully reflect clinical outcomes. Moreover, our study uses two commercially available SCC cell lines as a surrogate for AK, due to their shared basis in the pathway of keratinocyte carcinogenesis [30, 31]. Though SCC are widely used as a proxy to study AK, they represent a more advanced state of malignancy than AK. Another limitation lies in our exclusive use of 5‐ALA as the photosensitizing agent, rather than utilizing comparisons with MAL. MAL is lipophilic and may allow deeper intracellular penetration and accumulation, as compared to 5‐ALA, which is hydrophilic [11]. RL is FDA approved for Metvixia, a 16.8% MAL cream, and for Ameluz, a 10% ALA topical gel. [32, 33] The ALA used in the protocol is Levulan Kerastick, which is a 20% ALA topical solution that is FDA approved in combination with BL. [34] Finally, we did not directly quantify generation of ROS following PDT. Although ROS production is a key upstream mediator of PDT‐induced apoptosis, this study focused on downstream outcomes measured by Annexin V [23, 29]. Future studies are necessary to determine wavelength‐dependent differences in ROS generation and their correlation to apoptosis.

Future research can validate our findings in a novel AK‐derived cell line to better reflect the premalignant nature of AKs. Additionally, future studies can expand on these findings by investigating in vivo and ex vivo models and conducting clinical trials to examine light attenuation and quantify ROS production and cytotoxicity at clinically relevant tissue depths, as well as evaluate the contributions of vascular and immune mechanisms to PDT responses. Our results have important implications for optimizing PDT in the treatment protocol of AK and SCC. By enhancing our knowledge of the effects of light wavelength, 5‐ALA concentration, and temperature on PDT, therapy can be optimized to improve patient outcomes.

## 4 Conclusion

In summary, our data demonstrate that 5‐ALA BL PDT induces superior apoptosis in SCC cells in a wavelength‐, 5‐ALA dose‐, and temperature‐dependent manner, compared to 5‐ALA RL PDT, with higher concentrations of ALA and elevated temperatures stimulating the greatest apoptotic activity. These findings show promise for the optimization of PDT treatment for AK and early SCC lesions. Future research in human and animal models is necessary to corroborate these results.

## Funding

This work was supported by Sun Pharma.

## Conflicts of Interest

The VA received grant funding for this study. Dr. Jared Jagdeo has served as a scientific advisory board member for Sun Pharmaceuticals' educational content. All other authors have no conflicts of interest to disclose.

## Data Availability

The data that support the findings of this study are available from the corresponding author upon reasonable request.

## References

[1] E. Marques and T. M. Chen , “Actinic Keratosis,” in StatPearls. Treasure Island (FL) (StatPearls Publishing; August 17, 2023). 32491333

[2] C. Morton , R. M. Szeimies , A. Sidoroff , et al., “European Dermatology Forum Guidelines on Topical Photodynamic Therapy,” European Journal of Dermatology 25, no. 4 (2015): 296–311, 10.1684/ejd.2015.2570. 26065545

[3] A. Fuchs and E. Marmur , “The Kinetics of Skin Cancer: Progression of Actinic Keratosis to Squamous Cell Carcinoma,” Dermatologic Surgery 33, no. 9 (2007): 1099–1101, 10.1111/j.1524-4725.2007.33224.x. 17760601

[4] C. D. George , T. Lee , L. M. Hollestein , M. M. Asgari , and T. Nijsten , “Global Epidemiology of Actinic Keratosis in the General Population: A Systematic Review and Meta‐Analysis,” British Journal of Dermatology 190, no. 4 (2024): 465–476, 10.1093/bjd/ljad371. 37890083

[5] A. N. Kauvar , C. J. Arpey , G. Hruza , S. M. Olbricht , R. Bennett , and B. H. Mahmoud , “Consensus for Nonmelanoma Skin Cancer Treatment, Part II: Squamous Cell Carcinoma, Including a Cost Analysis of Treatment Methods,” Dermatologic Surgery 41, no. 11 (2015): 1214–1240, 10.1097/DSS.0000000000000478. 26445288

[6] R. Lucas , T. McMichael , W. Smith , B. K. Armstrong , A. Prüss‐Üstün , and Organization WH , Solar Ultraviolet Radiation: Global Burden of Disease From Solar Ultraviolet Radiation (World Health Organization, 2006).

[7] G. P. Guy, Jr. , S. R. Machlin , D. U. Ekwueme , and K. R. Yabroff , “Prevalence and Costs of Skin Cancer Treatment in the U.S., 2002‐2006 and 2007‐2011,” American Journal of Preventive Medicine February 48, no. 2 (2015): 183–187, 10.1016/j.amepre.2014.08.036. 25442229 PMC4603424

[8] M. V. Neidecker , M. L. Davis‐Ajami , R. Balkrishnan , and S. R. Feldman , “Pharmacoeconomic Considerations in Treating Actinic Keratosis,” PharmacoEconomics 27, no. 6 (2009): 451–464, 10.2165/00019053-200927060-00002. 19640009

[9] J. Yoon , J. Yoon , C. S. Phibbs , A. Chow , H. Pomerantz , and M. A. Weinstock , “Costs of Keratinocyte Carcinoma (Nonmelanoma Skin Cancer) and Actinic Keratosis Treatment in the Veterans Health Administration,” Dermatologic Surgery 42, no. 9 (2016): 1041–1047. 27465252 10.1097/DSS.0000000000000820

[10] J. Y. Wang , N. Zeitouni , E. Austin , J. Jagdeo , H. W. Lim , and D. M. Ozog , “Photodynamic Therapy: Clinical Applications in Dermatology,” Journal of the American Academy of Dermatology 20 (2025), 10.1016/j.jaad.2024.12.050. 39986392

[11] D. M. Ozog , A. M. Rkein , S. G. Fabi , et al., “Photodynamic Therapy: A Clinical Consensus Guide,” Dermatologic Surgery 42, no. 7 (2016): 804–827, 10.1097/DSS.0000000000000800. 27336945

[12] A. Huang , J. K. Nguyen , E. Austin , A. Mamalis , and J. Jagdeo , “Updates on Treatment Approaches for Cutaneous Field Cancerization,” Current Dermatology Reports 8, no. 3 (2019): 122–132, 10.1007/s13671-019-00265-2. 31475077 PMC6716609

[13] L. B. Josefsen and R. W. Boyle , “Photodynamic Therapy and the Development of Metal‐Based Photosensitisers,” Metal‐Based Drugs 2008 (2008): 276109, 10.1155/2008/276109. 18815617 PMC2535827

[14] Y. Ohgari , Y. Nakayasu , S. Kitajima , et al., “Mechanisms Involved in Delta‐Aminolevulinic Acid (ALA)‐induced Photosensitivity of Tumor Cells: Relation of Ferrochelatase and Uptake of ALA to the Accumulation of Protoporphyrin,” Biochemical Pharmacology December 19 71, no. 1–2 (2005): 42–49, 10.1016/j.bcp.2005.10.019. 16288996

[15] P. Gholam , I. Bosselmann , A. Enk , and C. Fink , “Impact of Red Versus Blue Light on Tolerability and Efficacy of PDT: A Randomized Controlled Trial,” Journal der Deutschen Dermatologischen Gesellschaft 16, no. 6 (2018): 711–717, 10.1111/ddg.13545. 29873905

[16] V. A. Patel , S. T. Arron , B. Berman , et al., “Expert Consensus‐Based Recommendations on the Use of Photodynamic Therapy in Actinic Keratosis Patients,” JAAD International 20 (2025): 62–73, 10.1016/j.jdin.2024.11.011. 40958824 PMC12433913

[17] H. Masuda , M. Kimura , A. Nishioka , H. Kato , and A. Morita , “Dual Wavelength 5‐Aminolevulinic Acid Photodynamic Therapy Using a Novel Flexible Light‐Emitting Diode Unit,” Journal of Dermatological Science February 93, no. 2 (2019): 109–115, 10.1016/j.jdermsci.2018.12.006. 30704937

[18] T. Hatakeyama , Y. Murayama , S. Komatsu , et al., “Efficacy of 5‐Aminolevulinic Acid‐Mediated Photodynamic Therapy Using Light‐Emitting Diodes in Human Colon Cancer Cells,” Oncology Reports 29, no. 3 (2013): 911–916, 10.3892/or.2013.2220. 23291627 PMC3597538

[19] C. Ash , M. Dubec , K. Donne , and T. Bashford , “Effect of Wavelength and Beam Width on Penetration in Light‐Tissue Interaction Using Computational Methods,” Lasers in Medical Science 32, no. 8 (2017): 1909–1918, 10.1007/s10103-017-2317-4. 28900751 PMC5653719

[20] A. J. Ruiz , E. P. M. LaRochelle , K. S. Samkoe , M. S. Chapman , and B. W. Pogue , “Effective Fluence and Dose at Skin Depth of Daylight and Lamp Sources for PpIX‐Based Photodynamic Therapy,” Photodiagnosis and Photodynamic Therapy 41 (2023): 103260, 10.1016/j.pdpdt.2022.103260. 36627070

[21] S. J. Martin , C. P. Reutelingsperger , A. J. McGahon , et al., “Early Redistribution of Plasma Membrane Phosphatidylserine Is a General Feature of Apoptosis Regardless of the Initiating Stimulus: Inhibition by Overexpression of Bcl‐2 and Abl,” Journal of Experimental Medicine 182, no. 5 (1995): 1545–1556, 10.1084/jem.182.5.1545. 7595224 PMC2192182

[22] A. S. Vignion‐Dewalle , N. Betrouni , J. B. Tylcz , M. Vermandel , L. Mortier , and S. Mordon , “Comparison of Three Light Doses in the Photodynamic Treatment of Actinic Keratosis Using Mathematical Modeling,” Journal of Biomedical Optics 20, no. 5 (2015): 58001, 10.1117/1.JBO.20.5.058001. 26000797

[23] L. Wang , V. S. Chelakkot , N. Newhook , S. Tucker , and K. Hirasawa , “Inflammatory Cell Death Induced by 5‐Aminolevulinic Acid‐Photodynamic Therapy Initiates Anticancer Immunity. *Front* ,” Oncologia 13 (2023): 1156763, 10.3389/fonc.2023.1156763. PMC10581343 37854679

[24] A. Mamalis , E. Koo , G. D. Sckisel , D. M. Siegel , and J. Jagdeo , “Temperature‐Dependent Impact of Thermal Aminolaevulinic Acid Photodynamic Therapy on Apoptosis and Reactive Oxygen Species Generation in Human Dermal Fibroblasts,” British Journal of Dermatology 175, no. 3 (2016): 512–519, 10.1111/bjd.14509. 26931503

[25] E. Koo , E. Austin , A. Mamalis , and J. Jagdeo , “Efficacy of Ultra Short Sub‐30 Minute Incubation of 5‐Aminolevulinic Acid Photodynamic Therapy in Vitro,” Lasers in Surgery and Medicine 49, no. 6 (2017): 592–598, 10.1002/lsm.22648. 28370019

[26] N. Schary , B. Novak , L. Kämper , A. Yousf , and H. Lübbert , “Identification and Pharmacological Modification of Resistance Mechanisms to Protoporphyrin‐Mediated Photodynamic Therapy in Human Cutaneous Squamous Cell Carcinoma Cell Lines,” Photodiagnosis and Photodynamic Therapy 39 (2022): 103004, 10.1016/j.pdpdt.2022.103004. 35811052

[27] S. C. Kanick , S. C. Davis , Y. Zhao , et al., “Pre‐Treatment Protoporphyrin IX Concentration in Actinic Keratosis Lesions May Be a Predictive Biomarker of Response to Aminolevulinic‐Acid Based Photodynamic Therapy,” Photodiagnosis and Photodynamic Therapy 12, no. 4 (2015): 561–566, 10.1016/j.pdpdt.2015.10.006. 26480810 PMC4684466

[28] N. Kibbi , Y. Zhang , D. J. Leffell , and S. R. Christensen , “Photodynamic Therapy for Cutaneous Squamous Cell Carcinoma in Situ: Impact of Anatomic Location, Tumor Diameter, and Incubation Time on Effectiveness,” Journal of the American Academy of Dermatology 82, no. 5 (2020): 1124–1130, 10.1016/j.jaad.2019.10.079. 31712171

[29] D. E. Dolmans , D. Fukumura , and R. K. Jain , “Photodynamic Therapy for Cancer,” Nature Reviews. Cancer 3, no. 5 (2003): 380–387, 10.1038/nrc1071. 12724736

[30] S. R. Feldman and A. B. Fleischer, Jr. , “Progression of Actinic Keratosis to Squamous Cell Carcinoma Revisited: Clinical and Treatment Implications,” Cutis 87, no. 4 (2011): 201–207. 21644496

[31] P. Bailey , R. A. Ridgway , P. Cammareri , et al., “Driver Gene Combinations Dictate Cutaneous Squamous Cell Carcinoma Disease Continuum Progression,” Nature Communications 14, no. 1 (2023): 5211, 10.1038/s41467-023-40822-9. PMC10457401 37626054

[32] Biofrontera, Inc ., “ *Ameluz (aminolevulinic acid hydrochloride) 10% gel* [package insert],” U.S. Food & Drug Administration (2024), https://www.accessdata.fda.gov/drugsatfda_docs/label/2024/208081s028lbl.pdf.

[33] Galderma A/S , “ *Metvixia (Methyl Aminolevulinate) Cream* [Package Insert]. U.S,” Food & Drug Administration (2012), https://www.accessdata.fda.gov/drugsatfda_docs/label/2012/021415s004lbl.pdf.

[34] DUSA Pharmaceuticals, Inc ., “ *Levulan Kerastick (Aminolevulinic Acid HCl) Kit* [Package Insert],” U.S. Food & Drug Administration (1999), https://www.accessdata.fda.gov/drugsatfda_docs/label/1999/20965lbl.pdf.

